# Supplementary material for: Measuring and explaining inequality of continuous care for people living with HIV receiving antiretroviral therapy in Kunming, China
Source: PLoS One. 2021 May 11;16(5):e0251252. doi: 10.1371/journal.pone.0251252 (PMC8112695; doi:10.1371/journal.pone.0251252)
Supplement: S1 File — (DOCX) [file pone.0251252.s001.docx]

## Structured questionnaire for people living with HIV.

| **Code** | **City** | **County/district** | **ARTID** |
| --- | --- | --- | --- |
|  |  |  |  |

Investigate time(yy/mm/dd): [ ] [ ] [ ] [ ] -[ ] [ ] - [ ] [ ]

**Section 1 Individual’s demographic characteristics and social factors**

| **Important question** | Duration of ART | ________years |
| --- | --- | --- |

| **A. Demographic characteristics** | | |
| --- | --- | --- |
| A1 | Your gender? | 1. Male 2. Female |
| A2 | How old are you? (completed year) | ___________years |
| A3 | Your weight in Kilos? | ___________Kilos |
| A4 | Your height in Meters? | ___________Meter |
| A5 | What is your ethnicity? | 1.Han 2. Yi 3. Hui  4. Bai 5. Miao 6. Hani  7.Dai 8. Zhuang 9. Other |
| A6 | What is your religion? | 1. No 2. Buddhism 3. Christianity  4. Islam 5. Others |
| A7 | What is your marital status? | 1. Never married 2. Currently married  3.Separated 4. Divorced  5.Widowed 6. Cohabiting |
| **B** | **Socio-economic characteristics** |  |
| B1 | Number of family members (include yourself) | ____________ |
| B2 | Number of family members aged 14 years and under | ____________ |
| B3 | Do you own a house of apartment? | 1. Yes 2. No |
| B4 | How many square meters of your dwelling/house? | _____________mm^2^ |
| B5 | How many bedrooms (including guest bedrooms, bedrooms used as offices etc.) are in your dwelling/house? | _____________ |
| B6 | What is type of floor in your dwelling/house? | 1.Hard floor (tile, cement, brick, wooden floor) 2. Earth floor |
| B7 | What is main source of drinking water in your dwelling/house? | 1.Piped water through house connection or yard  2. Public standpipe  3. Protected tube well or bore hole  4. Protected dug well or protected spring  5. Unprotected dug well or spring  6. Rainwater (into tank or cistern)  7. Water taken directly from pond-water or stream  8. Tanker-truck, vendor |
| B8 | What is type of toilet facilities in your dwelling/house? | 1. Flush to piped sewage system.  2. Flush to specific tank.  3.Pour flush latrine.  4. Other |
| B10 | Does your household own the following appliances? | |
| B10.1 | Television | 1. Yes 2. No |
| B10.2 | Refrigerator | 1. Yes 2. No |
| B10.3 | Washing machine | 1. Yes 2. No |
| B10.4 | Computer | 1. Yes 2. No |
| B10.5 | Microwave | 1. Yes 2. No |
| B10.6 | Mobile phone | 1. Yes 2. No |
| B10.6 | Internet broadband | 1. Yes 2. No |
| B11 | Does your household own the following furniture? | |
| B11.1 | Table | 1. Yes 2. No |
| B11.2 | Chair | 1. Yes 2. No |
| B11.3 | Sofa | 1. Yes 2. No |
| B11.4 | Bed | 1. Yes 2. No |
| B11.5 | Wardrobe | 1. Yes 2. No |
| B11.6 | cupboard | 1. Yes 2. No |
| B9 | What is main type of fuel for cooking in your dwelling/house? | 1. Gas  2. Electricity  3. Kerosene  4. Coal  5. Wood  6. Charcoal  7. Agriculture/crop  8. Animal dung  9. Shrubs/grass  10.Others |
| B12 | Dose your household OWN a motor vehicle (car, truck, van, SUV) now? | 1.Yes 2. No  If yes, how many do you own? __________________________ |
| C | What is the highest level of education that you have completed? | 1.No formal schooling  2.Less than primary school  3.Primary school completed  4.Secondary school completed  5. High school (or equivalent) completed 6. College/University completed or above |
| E1 | What is your employment status? | 1. Employed 2. Retired  2. No job |
| E2 | What is your current job? | 1. Government employee  2. Non-government employee  3. Self-employed  4. Employer  5. Not working for pay |
| L1 | Health insurance status | 1. The new rural cooperative medical system  2. Urban employee basic medical insurance,  3. Urban residence basic medical insurance,  4. uninsured. |

**Perceived mental health care:**

| K3 | Have you received a mental health assessment from a health provider in the last 12 months? | 1. Yes 2. No |
| --- | --- | --- |

**Thank you for your participation!**

# Medical record review form (ARTID[ ])

| Date of data review ________________(yy/mm/dd) | |
| --- | --- |
| Reviewer ________________ | |
| Date of HIV diagnosis | / / (yy/mm/dd) |
| Date of ART initiation | / / (yy/mm/dd) |
| Initiated ART regimen |  |
| Type of ART | 1. Free 2. Part of free 3. Out-of-pocket. |
| Recent ART regimen |  |
| Number of visits during the last 6 months |  |
| Latest CD4 count | ________________cells/ul |
| Number of CD4 test in the last 12 months | _______________ |
| Latest viral load | ________________copies/ml |
| Number of a viral load test in the last 12 months | _______________ |
| Number of receiving routine test package (liver, renal function, and blood cell count) | _______________ |

| **编码** | **城市** | **区/县** | **ARTID** |
| --- | --- | --- | --- |
|  |  |  |  |

**获得性免疫缺陷综合征患者获得医学健康服务及相关因素的调查**

调查日期（年/月/日）: [ ] [ ] [ ] [ ] -[ ] [ ] - [ ] [ ]

| **重要问题** | 抗病毒治疗持续时间 | ________年 | selfartduration[ ] |
| --- | --- | --- | --- |

| A. 人口学特征 | | | CODE |
| --- | --- | --- | --- |
| A1 | 性别 | 1. 男 2.女 | sex[ ] |
| A2 | 您的年龄？ | ___________岁 | age[ ][ ] |
| A5 | 您的民族？ | 1.汉族2.彝族3.回族4.白族5.苗族 6.哈尼族7.傣族 8.壮族9.其他 | ethnic[ ] |
| A6 | 您的宗教信仰？ | 1.没有2.佛教3.基督教4.伊斯兰教5.其他 | religion[ ] |
| A7 | 您的婚姻状况？ | 1.没有结过婚 2.已婚4.离异5.鳏寡 6.同居 | marrige[ ] |

**第一部分: 患者获得医疗服务**

| K3 | 最近一年您接受过医务人员给您做心理健康状况的评估吗？ | 1.是 2.否 | evadepress[ ] |
| --- | --- | --- | --- |

**第二部分：患者的社会经济学特征**

| B | **家庭规模及经济情况** | | |
| --- | --- | --- | --- |
| B1 | 家庭成员的数量（包括你自己） | ____________人 | numhh[ ][ ] |
| B2 | 年龄14岁及以下家庭成员的数量 | **_____________**人 | childhh[ ] |
| B5 | 您有自己的房子或者公寓吗？ | 1. 有 2. 无 | house[ ] |
| B7 | 您的住房有多少平米？ |  | shs[ ][ ][ ] |
| B8 | 您的住所有多少间卧室？ | _____________ | bedroom[ ] |
| B9 | 您的住所/房子的地板是什么类型的？ | 1. 硬地板（瓦、水泥、磁砖、木地板）  2.土地面 | floor[ ] |
| B10 | 您的家庭饮用水的主要来源？ | 1.公用自来水2.井水3.桶装纯净水  4.保护好的或挖好的泉水  5.直接取自池塘或溪流的水 | water[ ] |
| B11 | 您的房子使用什么类型的厕所设施？ | 1.马桶/蹲厕冲进管道的污水系统  2. 马桶或者蹲厕冲进特殊的蓄粪池  3.自己倒入公共厕所4.其他 | toilet[ ] |
| B12 | 您的家庭是否有以下家电设备？ | 电视机 1.有 2. 无  冰箱 1.有 2. 无  洗衣机 1.有 2. 无  电脑 1.有 2. 无  微波炉 1.有 2. 无  电话（固定电话和手机）1.有 2. 无  宽带 1.有 2. 无 | tv[ ]  rf[ ]  wm[ ]  cp[ ]  mw[ ]  tp[ ]  wlv[ ] |
| B11 | 您的家庭是否有以下家具？ | 桌子 1.有 2. 无  椅子 1.有 2. 无  沙发 1.有 2. 无  床 1.有 2. 无  衣柜 1.有 2. 无  橱柜 1.有 2. 无 | tab[ ]  cha[ ]  saf[ ]  bed[ ]  wad[ ]  cab[ ] |
| B12 | 您的家庭煮饭的**主要**燃料是哪些？ | 1.天然气2.电3.煤油4.煤5.木头 6.木炭7.农作物8.动物粪便9.灌木、草10.其他___________ | fuel[ ] |
| B13 | 您属于自己的机动车（轿车、卡车、面包车、SUV）吗？ | 1.是 2.否  如果有，你有多少辆车？________ | car[ ]  carnum[ ] |
| **C. 教育情况** | | |  |
| C | 您完成的最高教育水平？ | 1.无正式教育2.低于小学3.小学4.中学  5.高中(同等学历如职高、中专)6.大学或以上 | edu[ ] |
| **E.您的职业和工作环境** | | |  |
| E1 | 您的就业情况？ | 1.在职2退休 3.无业 | job[ ] |
| E2 | 您目前的工作类型？ | 1. 政府公务员（事业单位）2.非政府的一般雇员3.个体4. 不稳定职业5.不为薪水而工作（自由职业） | jobtype [ ] |
|  | | |  |
| **3.您的医学保健环境** | | |  |
| L1 | 您的医疗保险状态(三项基本国家医疗保险计划之一) | 1.新农合系统  2.城镇职工基本医保  3.城镇居民基本医疗保险  4.没有医保 | hcinsurane[ ] |

**谢谢您的帮助！**

**医疗记录数据收集(抗病毒治疗号[ ]）**

浏览日期 _______________________ (年/月/日) 浏览者____________________

| R1 | 婚姻状况 | 1.没有结过婚2.已婚或同居3.单身 4.离异5.鳏寡 6.同居 | marri[ ] |
| --- | --- | --- | --- |
| R2 | 确诊HIV时间 | / / （年/月/日) | hivdate[ ] |
| R3 | 抗病毒开始日期 | / / （年/月/日) | artdate[ ] |
| R7 | 初始ART方案 |  | artre1[ ] |
| R8 | 抗病毒治疗类型 | 1.免费2.部分自费 3.全自费 | artty[ ] |
| R9 | 目前ART方案 |  | artre2[ ] |
| R10 | 最近6个月来诊次数 |  | visit [ ] |
| R12 | 最近VL(cps/ml) |  | vln [ ] |
| R13 | VL最近一年检测次数 |  | vlt [ ] |
| R15 | 最近CD4计数(个/mm^3^) |  | cd4n [ ] [ ] [ ] [ ] |
| R16 | CD4最近一年检测次数 |  | cd4times[ ] |
| R17 | 耐药 | 1.是 2.否 3. 未测 | artresist[ ] |
| R18 | 最近一年常规实验室检测次数(肝肾功血常规) | ________次 | artrt[ ] |
