# Supplementary material for: Measuring and explaining inequality of continuous care for people living with HIV receiving antiretroviral therapy in Kunming, China
Source: PLoS One. 2021 May 11;16(5):e0251252. doi: 10.1371/journal.pone.0251252 (PMC8112695; doi:10.1371/journal.pone.0251252)
Supplement: Attachment — Submitted filename: Responses to Reviewers.docx [file pone.0251252.s004.docx]

Responses to Editor and Reviewer

PLOS One Manuscript number: PONE-D-20-28608

Measuring and explaining inequality of continuous care for people living with

HIV on antiretroviral therapy in Kunming, China

Additional Editor Comments:

1.Overall, the quality of writing left the reviewers in difficulty judging the study. Please follow standard scientific report writing rules (e.g., use of tenses and terminologies, spelling out numbers smaller than 10, use of tenses and punctuations). I have spotted several easy grammatical errors, typos, and complex sentences across the manuscript. Many statements also require supporting references and clarification. In the text, please minimize the use of uncommon and unnecessary abbreviations (IRC, RTM, MIO, MVO, AMH, etc.) that reduce the manuscript’s readability. The manuscript is not publishable in its current form and will benefit from thorough proofreading by a native or more experienced academic writer. Below are some suggestions, which may not be exhaustive. We will decide whether to continue reviewing the paper based on the improvement made in the next submission.

Response: Thank you for your valuable comments. We have checked and made revisions accordingly. We have also asked a native English academic to edit the final manuscript.

2.Title and keywords:

- Please revise the title according to PLOS’ styles and guidelines (e.g., do not start each word with a capital).

Response: Checked and revised accordingly.

- Please remove ‘AIDS’ from ‘people living with HIV.’ UNAIDS recommended that ‘HIV/AIDS’ should not be used since 2015. Also, ‘people living with HIV’ should not be abbreviated or addressed as patients. This is applied across the paper, including keywords. See: <https://www.unaids.org/sites/default/files/media_asset/2015_terminology_guidelines_en.pdf>

Response: Thank you for your advice. We have checked and made revisions accordingly.

- To increase the visibility of your paper, please avoid keywords that are overlapping with those in the title. This may help: <https://falconediting.com/en/blog/6-tips-for-choosing-keywords-for-your-scientific-manuscript>

Response: Thank you for your suggestions. We have modified the keywords as: HIV, healthcare utilization, healthcare inequality, concentration index, decomposition, China.

- For PLOS journals, keywords should be included in the submission system, not in the text.

Response: We have made revisions accordingly.

3. Abstract:

- A structured abstract is recommended (background, methods, results, conclusions).

Response: We have made revisions accordingly.

- In Methods, please provide the study period and summarize the sampling methods.

Response: We have made revisions accordingly.

- Results should provide the sample size and essential characteristics (age, sex).

Response: We have added the sample size and essential characteristics (age, sex) in the methods part.

4. Introduction:

- The Introduction contains long and complex sentences that are difficult to read and need to be restructured.

Response: We have rewritten the introduction part.

5. Materials and Methods:

- Inclusion criteria should be revised to improve the quality of writing and understanding and avoid redundancy. For example, the first sentence under the ‘Study setting and participants’ stated that this study included people living with HIV who had initiated ART for at least one year, but not more than five years. This information was repeated in inclusion criteria 3, and it was not entirely consistent.

Response: We have made revisions accordingly.

(Lines 152-156: “Individuals were eligible to be included in the study if they: 1) were aged 18 years or older; 2) had HIV infection clinically diagnosed and confirmed with serological tests; 3) had initiated ART and were currently receiving ART for more than 1 year but not more than 5 years; 4) were able and willing to provide verbal consent to participate in the study, and; 5) were able to communicate in Chinese.”)

- Was sample size calculation performed?

Response: Yes, we have added the sample size calculation in the revised manuscript.

(Lines 164-170: “The sample size was calculated using the finite population formula. As no previous data on comprehensive care access among PLWH was available in China, it is conservatively estimated that comprehensive care access was 50%, there were nearly 8,000 people receiving ART in the HIV treatment center, considering a margin of error of 4%, a confidence interval of 95% and a 20% refusal rate or missing data. Finally, the minimum required sample size for the study was 664.”)

- Please provide a reason for excluding people living with HIV aged 18 and those who had received ART more than five years.

Response: We did not exclude people living with HIV aged 18 years. We apologize for the confusing sentence in the previous version. In this version, we have rewritten the inclusion criteria to make them clearer. (Lines 152-153)

- Inclusion criteria 3 is difficult to read, and criteria 5 is not understandable. What does ‘physical and psychological ability’ mean? What happened if someone could not complete the questionnaire in 30 minutes?

Response: We have checked and made revisions accordingly. We have changed “physical and psychological ability” to “were able and willing to provide verbal consent to participate in the study and were able to communicate in Chinese”. (Lines 155-156)

- Please provide references to support variable measurements. I am not quite sure if the criteria remained applicable in China in 2019. For example, are people living with HIV are still required to visit an ART clinic two times or more in 90 days? Is mental health a component in the treatment and care service package?

Response: We have made revisions accordingly (Lines 196-224). People living with HIV were still required to visit the ART clinic every 3 months in 2019. Based on the free ART manual of China free antiretroviral treatment program (NFATP), after initiating ART for more than one-year, subsequent follow-up visits are scheduled every 3 months, and the dispensing of free ART is three-monthly in the HIV treatment center to ensure enough stocks of ART for both newly initiated and continuously treated people. Mental health is not a standard component in the ART service package. However, Chinese national guidelines recommend that mental healthcare can be integrated into ART services when conditions permit, but there are no specific operating guidelines or records in the outpatient system or database of NFATP.

- Please be sensitive in using the term ‘gender’ and ‘sex.’ Please double-check if ‘gender’ is the correct term for this study context.

Response: We have checked and made revisions accordingly. We have used “sex” or “biological sex” throughout the manuscript.

- Line 163: What does ‘need or non-need factor’ mean?

Response: As stated in the Introduction (lines 131-135) and Materials and methods (Lines 233-235), “Possible determinants (of healthcare inequality) can be categorized into “need” and “non-need” factors. Need factors are sources of variation in healthcare that are not considered to be “unfair”. They are related to characteristics of an individual that are direct causes of the use of medical services, usually age and sex. Non-need factors are sources of variation in health care that are “unfair” or “unjust”, such as SES, education, and ethnicity.” In this study, “need” factors include age and sex and other socio-demographic factors are “non-need” factors.

- Data analysis needs further details and should be aligned with the result presentation. For example, I am not sure if I understand why the authors compare socio-demographic characteristics in SES groups (Table 1), which is quite predictable. In the data analyses, the authors mentioned about the comparison of dependent variables in different groups. It was also not clear whether the analyses were controlled for confounding factors. If so, how were the models built, or how were the covariates selected? Only Bonferroni's adjustment was mentioned.

Response: We have revised Table 1 to show the distribution and concentration indices of socio-demographic characteristics and reduced comparison of SES groups; we employed the Stata command “conindex” to calculate the p-value for testing if the concentration index is equal to zero. Because our outcomes were binary, the decomposition of the concentration index involved using a probit model with marginal effects for all socio-demographic explanatory variables. We only focused on how socio-demographic explanatory variables influenced healthcare inequalities, so we did not include other health variables and clinical factors and reduced the socio-demographic variables from the model.

- Ethics: Describe the measures taken to protect participants’ privacy and confidentiality. Also, did the participants receive any compensation?

Response: We have made revisions accordingly. (Lines 174-176, 179-182 and 338-345). Because we conducted the study when participants were attending the routine visits, there was no compensation given to them.

6. Results

- The wording and terminologies used in the description of the results need improvement.

Response: We have checked and made revisions accordingly. We have rewritten several parts of the results to make them clearer and easier to read.

- Descriptive results can be summarized as they are mostly repeating the information in tables.

Response: We have made revisions accordingly.

- Table 2: Exact p values should be presented rather than showing *p-value <0.05, ** p-value <0.01, and ***p-value <0.001 in the footnote.

Response: We have made revisions accordingly. (Table 1 and Table 2 )

7. Discussion

- Please refine the discussion according to the earlier comments and reviewer’s comments, focusing on this study's significant findings. It is also important to discuss the socio-economic burden of HIV care continuum on people living with HIV, particularly in the study setting context (e.g., free vs. charged services, other costs).

Response: Thank you for your suggestion. We have revised the discussion part and focussed on the significant findings. (Line 448-475)

8. References

- The reference list needs to be revised to be aligned with PLOS’s styles and requirements.

Response: We have revised the reference style as “Vancouver” based on PLOS’s requirements.

Journal Requirements:

When submitting your revision, we need you to address these additional requirements.

1. Please ensure that your manuscript meets PLOS ONE's style requirements, including those for file naming. The PLOS ONE style templates can be found at

<https://journals.plos.org/plosone/s/file?id=wjVg/PLOSOne_formatting_sample_main_body.pdf> and

<https://journals.plos.org/plosone/s/file?id=ba62/PLOSOne_formatting_sample_title_authors_affiliations.pdf>

Response: We have made revisions accordingly.

2. Please include additional information regarding the survey or questionnaire used in the study and ensure that you have provided sufficient details that others could replicate the analyses. For instance, if you developed a questionnaire as part of this study and it is not under a copyright more restrictive than CC-BY, please include a copy, in both the original language and English, as Supporting Information, or include a citation if it has been published previously.

Response: We have added accordingly.

3. Please include captions for your Supporting Information files at the end of your manuscript, and update any in-text citations to match accordingly. Please see our Supporting Information guidelines for more information: <http://journals.plos.org/plosone/s/supporting-information>.

Response: We have added and update accordingly.

Reviewers' comments:

1. Is the manuscript technically sound, and do the data support the conclusions?

The manuscript must describe a technically sound piece of scientific research with data that supports the conclusions. Experiments must have been conducted rigorously, with appropriate controls, replication, and sample sizes. The conclusions must be drawn appropriately based on the data presented.

Reviewer #1: Partly

2. Has the statistical analysis been performed appropriately and rigorously?

Reviewer #1: Yes

3. Have the authors made all data underlying the findings in their manuscript fully available?

The [PLOS Data policy](http://www.plosone.org/static/policies.action#sharing) requires authors to make all data underlying the findings described in their manuscript fully available without restriction, with rare exception (please refer to the Data Availability Statement in the manuscript PDF file). The data should be provided as part of the manuscript or its supporting information, or deposited to a public repository. For example, in addition to summary statistics, the data points behind means, medians and variance measures should be available. If there are restrictions on publicly sharing data—e.g. participant privacy or use of data from a third party—those must be specified.

Reviewer #1: No

4. Is the manuscript presented in an intelligible fashion and written in standard English?

PLOS ONE does not copyedit accepted manuscripts, so the language in submitted articles must be clear, correct, and unambiguous. Any typographical or grammatical errors should be corrected at revision, so please note any specific errors here.

Reviewer #1: No

Response: We have made revisions accordingly.

5. Review Comments to the Author

Please use the space provided to explain your answers to the questions above. You may also include additional comments for the author, including concerns about dual publication, research ethics, or publication ethics. (Please upload your review as an attachment if it exceeds 20,000 characters)

Reviewer #1:

1) Many concentration indices were with non-significant p-values, but the authors still interpreted as if there were effect, which is inappropriate. The results of the manuscript should be summarized in a succinct manner with the significant and important results stressed.

Response: Thank you for your suggestion. We have revised the discussion part and focussed on the significant findings. (Line 448-475)

2) Table 3, it is unclear to me for the same variable why the absolute contribution and percentage of contribution to concentration index can have different signs?

Response: As we described in lines 282-285 and 293-297, the percentage of contribution equals (100*(*β_k_ ̅x_k_* / *μ*) *C_k_/ CI_n_)*. If the concentration index is negative, the absolute contribution can follow the concentration index sign as a negative value, but the contribution percentage was positive, so the positive contribution percentage increases the size of the inequality, a negative contribution percentage decreases the size of the inequality.

3) Please ask for professional editing help with English language.

Response: Thank you. We have asked for professional editing help with the English.
