# Supplementary material for: Measuring and explaining inequality of continuous care for people living with HIV receiving antiretroviral therapy in Kunming, China
Source: PLoS One. 2021 May 11;16(5):e0251252. doi: 10.1371/journal.pone.0251252 (PMC8112695; doi:10.1371/journal.pone.0251252)
Supplement: Attachment — Submitted filename: Rebuttal letter.docx [file pone.0251252.s005.docx]

Rebuttal letter to Editor and Reviewer

PLOS One Manuscript number: PONE-D-20-28608

Measuring and explaining inequality of continuous care for people living with HIV receiving antiretroviral therapy in Kunming, China

Journal Requirements:

Please review your reference list to ensure that it is complete and correct. If you have cited papers that have been retracted, please include the rationale for doing so in the manuscript text, or remove these references and replace them with relevant current references. Any changes to the reference list should be mentioned in the rebuttal letter that accompanies your revised manuscript. If you need to cite a retracted article, indicate the article’s retracted status in the References list and also include a citation and full reference for the retraction notice.

Response: As we condensed the introduction, we removed six references and revised the reference list.

Additional Editor Comments:

We thank the authors for addressing the reviewers’ comments. The revised manuscript has been greatly improved. However, it still requires further attention, particularly in the Introduction and Methods. Below are some suggestions, that may not be exhaustive. The revised manuscript requires thorough proofreading to remove several minor grammatical errors, typos, terminology use, tenses, etc. Also, please ensure that your revised manuscript is prepared according to the journal’s styles and guidelines, particularly in the reference list. Please note that PLOS usually does not provide an opportunity for the authors to proofread their paper before the publication if it is accepted.

Response: Thank you for your valuable comments. We have checked and made revisions accordingly, especially Introduction and Methods. We have checked and made revisions in the reference list based on the journal’s styles and guidelines.

Title page:

1. Please use a consistent font and font size.

Response: We have checked and made revisions accordingly.

Abstract:

2. As commented earlier ‘people living with HIV’ should not be abbreviated or addressed as patients. This is applied across the paper, including keywords. See: <https://www.unaids.org/sites/default/files/media_asset/2015_terminology_guidelines_en.pdf>

Response: We made revisions accordingly across the paper.

3. Please refine the objective of the study, which is too long and difficult to read. The authors may consider breaking it down into two sentences. Also, ‘impact’ is an incorrect term in this context, given the study’s cross-sectional nature.

Response: We have made revisions accordingly. (Page 2 lines 15-19: We have split the objectives into two sentences. Line 19: We changed “impact” to “contributions”.)

4. Results:

- Line 28: what does ‘uptakes of retention’ in care mean?

Response: We have modified this sentence accordingly. (Page 2 line 28: We removed “Uptakes of.”)

- Is there any other ways to explain ‘non-significant pro-rich and pro-poor,’ that are easier to understand?

Response: We have revised this as follows: (Page2 lines 29: We revised “non-significantly pro-rich” to “non-significant in favor of the rich” And lines 30-31: we revised “nonsignificant pro-poor” to “higher among the poor.”)

- Please make it clear if the last results (Lines 31-33) were statistically significant.

Response: We calculated contributions of socioeconomic status to inequalities based on equation (5), finally we did not check for statistical significance.

Introduction

5. Overall, the introduction is well written but unnecessarily too long that should be condensed. Please try to limit it to approximately 1000 words (1-2 paragraphs for problem statement, 1-2 paragraphs on literature review of previous studies on inequality in access to HIV services and its associated factors, and 1 paragraph on rationales, and 1 paragraph on study’s objectives).

Response: Thank you for your comments. We have checked revised the introduction accordingly.(Pages 3-6)

6. Please also proofread it carefully after the revisions (e.g., on line 45, no need to write ART in full as it was already spelled out on line 43)

Response: Checked and revised accordingly.

Materials and Methods

7. Line 150-151: It sounds a little bit awkward when saying, “Kunming city was chosen as the study area because it has the highest prevalence of HIV/AIDS in Yunnan.” Any rationale directly linked to Kunming? The prevalence of HIV also needs a reference to support. Please remove ‘AIDS’ from the highest prevalence of HIV/AIDS. As commented earlier, PLWH is not currently in use as recommended by UNAIDS.

Response: Revised accordingly.

Lines 116-117: As we have no rationale directly linked to Kunming, thus we have removed the sentence referring to Kunming city being chosen due to its high prevalence of HIV/AIDS and added: “Kunming is the capital city of Yunnan province.” We have also removed “AIDS” accordingly.

8. Eligibility criteria:

- Does HIV infection require a clinical diagnosis? Many people living with HIV do not present with any symptoms and are diagnosed only by serological tests – were they eligible for the study? Usually, getting registered to receive HIV services indicates that they live with HIV, which is clearly stated in criteria 3.

Response: Thank you for your suggestion. We did not require a clinical diagnosis for HIV infection. We have therefore removed this inclusion criteria.

- Any criteria related to physical and mental health stability to participate in the interview?

Response: Yes.

Page 6 line 122-124: We added the following criteria: “individuals were able to present themselves on the day of the interview and sufficiently physically and mentally stable to provide verbal consent to participate in the study” to participate in the study.

- Lines 154, 157, 159: As commented earlier, numbers smaller than 10 should be spelled out in words in scientific and academic writing. This is applied to the whole document.

Response: Revised accordingly.

- Line 155: Please remove ‘,’ before ‘and.’

Response: Done.

- Lines 156-161: The sentence is too long and should be broken down into 2-3 sentences.

Response: Done. (Pages 6-7 lines 124-130)

- Line 161: Please replace ‘;’ by. ‘’

Response: Done. (Page 7 line 130)

- Lines 165-169: The sentence is too long and should be broken down into 2 sentences.

Response: Done. (Page 7 lines 134-139)

9. Line 184: Please spell out NFATP if it was used for the first time in the manuscript.

Response: NFATP was spelled out in the Introduction section. (Page 4 line 66)

10. Line 192: Guess ‘outcome’ should read ‘outcome variable.’

Response: Yes - revised accordingly. (Page 8 line 161)

11. Line 197: Not sure if the word ‘free’ before ‘ART manual’ is necessary. Is the manual applied only for ‘free’ ART? I do not find it necessary to keep saying ‘free ART’ (e.g., lines 198, 201) as it brings more confusion than help. The authors may just describe in the description of the study site that the HIV center where the study was conducted provides ART and other HIV services free of charges.

Response: Thank you. On page 8 lines 167-171: The manual applied only for free-ART, the HIV center provides free ART, and other services are not free of charge. We have the manual to guide the free-ART services, listed in reference 20, and the Chinese national guideline to guide the national treatment and services for people living HIV, listed in reference 21.

12. Line 211: The use of ‘;’ before ‘and’ is incorrect.

Response: Thank you. Revised accordingly. (Page 9 line 181)

13. The NFATP only provides free CD4 and viral load testing once a year, and people living with HIV have to pay for the remaining tests. It is important to state whether the non-free tests were voluntary based on the clients’ ability to pay or compulsory as the test costs can be beyond their affordability. This may explain the relationships between inequality and socio-demographic characteristics, leaving alone other potential financial barriers (transport costs, busy working). I would suggest this point be discussed in the discussion section. This may also explain the low rate of viral load test presented in the results.

Response: Thank you for your valuable comments. We have made some revisions accordingly. (Page 9 lines 184, 188-189: the non-free tests were voluntary based on the client’s ability to pay, and test costs can be beyond their affordability. Page 24 lines 479-482: We added this point into the Discussion section.)

14. Line 256: Please use past tense in the methods section (e.g., this study used…).

Response: Done. (Page11 line 228)

15. Line 329: …using the Chi-square test.

Response: Thank you. (Page 14 line 303: SES groups were calculated and compared using the chi-square test.)

16. Line 352: Please present the median value with an interquartile range.

Response: Done. (Page 15 line 326: median age of 41.0 years (interquartile range, 32.0 to 48.0 years))

17. Line 496: WHO was already defined in the introduction’.

Response: Thanks and revised accordingly. (Page24 line 471)

18. Lines 497-498: the sentence “A study in Thailand suggested that simple and inexpensive monitoring of key biomarkers could be done at some time points” is not clear and should be further elaborated. What did the authors intend to tell – feasibility, efficacy, or else?

Response:We meant feasibility. (Page 24 lines 472-473: A study in Thailand demonstrated that the simple and inexpensive monitoring of key biomarkers was feasible at some time points)

Lines 523-524: A self-reporting measure can lead to both under- or over-reporting unless this study could prove that it was under-reporting.

Response: We cannot prove either way. (Page 26 lines 499-500: the household assets and conditions were self-reported, which might result in both under- or over-reporting of participants’ SES.)

20. Line 556: Guess Kunming does not require ‘the.’

Response: Yes, of course. Corrected.(Page 27 line 534)

21. Lines 556-559: Conclusions should summarize the key findings that respond to the research questions and objectives. The comparison of this study’s findings to the literature should be discussed in the discussion section.

Response: Thank you. Please see page 27 lines 534-537.

Lines 563-565: The sentence “Furthermore, need variables (i.e., age and sex) could not negatively contribute to all concentration indices to diminish the degree of inequalities” needs clarity as it is hard to understand.

Response: We have revised this as follows: (Page27 lines 541-544: Between the two need variables (age and sex), age contributed more to the inequalities in the utilization of all HIV continuous care. This implies that the degree of such inequalities can be reduced should comprehensive care be provided equally to people living with HIV of all age groups.)

23. References are still inconsistent and not entirely aligned with PLOS’s style and requirements. Please ensure that they have been corrected before re-submission.

Response: We have checked the references carefully and revised them accordingly. (Pages 29-33)
